# Comparative Study on the Fungicidal Activity of Metallic MgO Nanoparticles and Macroscale MgO Against Soilborne Fungal Phytopathogens

**DOI:** 10.3389/fmicb.2020.00365

**Published:** 2020-03-12

**Authors:** Juanni Chen, Lintong Wu, Mei Lu, Shasha Lu, Ziyan Li, Wei Ding

**Affiliations:** Laboratory of Natural Products Pesticide, College of Plant Protection, Southwest University, Chongqing, China

**Keywords:** magnesium oxide nanoparticle, fungicidal activity, soilborne fungus, disease management, direct interaction, *in vivo* study

## Abstract

Engineered nanoparticles have provided a basis for innovative agricultural applications, specifically in plant disease management. In this interdisciplinary study, by conducting comparison studies using macroscale magnesium oxide (mMgO), we evaluated the fungicidal activity of MgO nanoparticles (nMgO) against soilborne *Phytophthora nicotianae* and *Thielaviopsis basicola* for the first time under laboratory and greenhouse conditions. *In vitro* studies revealed that nMgO could inhibit fungal growth and spore germination and impede sporangium development more efficiently than could macroscale equivalents. Indispensably, direct contact interactions between nanoparticles and fungal cells or nanoparticle adsorption thereof were found, subsequently provoking cell morphological changes by scanning electron microscopy/energy-dispersive spectrometry (SEM/EDS) and transmission electron microscopy (TEM). In addition, the disturbance of the zeta potential and accumulation of various modes of oxidative stress in nMgO-exposed fungal cells accounted for the underlying antifungal mechanism. In the greenhouse, approximately 36.58 and 42.35% decreases in tobacco black shank and black root rot disease, respectively, could testify to the efficiency by which 500 μg/ml of nMgO suppressed fungal invasion through root irrigation (the final control efficiency reached 50.20 and 62.10%, respectively) when compared with that of untreated controls or mMgO. This study will extend our understanding of nanoparticles potentially being adopted as an effective strategy for preventing diversified fungal infections in agricultural fields.

## Introduction

Tobacco (*Nicotiana tabacum* L.), known as one of the most important economic crops in the world, has long suffered from several devastating soilborne bacterial and fungal diseases (Jiang et al., 2017). *P. nicotianae* and *Thielaviopsis basicola*, the causes of black shank and black root rot diseases, respectively, are regarded as two of the most catastrophic and widespread filamentous eukaryotic phytopathogenic oomycetes and are persistent threats to more than 50 plant species and damage the root and stem, reducing crop production (Parkunan et al., 2010). In view of the large, seasonally variable genetic diversity and highly virulent populations of these root-damaging soil organisms (Verniere et al., 2004; Park Y.J. et al., 2006; Alvarez et al., 2011), effective management practices, such as cultural practices, host resistance, crop rotation (Curl, 1963; Fang et al., 2016), biofungicides, and chemical control (Ji et al., 2014), are commonly adopted by managers. However, there remain some factors hindering permanent application. As scientific technology has progressed, it has become highly desirable to develop novel alternative approaches for managing soilborne fungal diseases.

Nanotechnology is increasingly exploited in a wide range of agricultural applications, including plant nutrition, soil remediation, pathogen detection, and disease and pest control (Khot et al., 2012), with the aim of a less-formulated product with a higher content of the active component than commercial agrochemicals. A series of inorganic and organic nanomaterials have been developed and proven to exhibit prominent antibacterial, antifungal, and antiviral properties on phytopathogenic microbes *in vitro*, and some of them still exerted their toxicity effects under greenhouse and field conditions. To date, TiO_2_, CuO (Hao et al., 2017, 2019; Liu et al., 2017), Zn, ZnO (Xue et al., 2014; Antonoglou et al., 2018; Sun et al., 2018), carbon nanomaterials (Chen et al., 2014, 2016b), Al, and Si nanoparticles (Park H.J. et al., 2006; Shenashen et al., 2017) have been reported to display toxicity toward phytopathogenic bacteria and fungi, decreasing the disease incidence. Recently, our group also found that graphene oxide silver nanoparticle (GO-AgNP) nanocomposites can suppress the development of hyphae, showing a significant effect in controlling leaf spot disease from *Fusarium graminearum* (Chen et al., 2016b). The high-efficiency antimicrobial activity of these nanocomposites is generally ascribed to their superior physicochemical properties, high surface-to-volume ratio, and unique nanoscale structural characteristics (Stoimenov et al., 2002).

Magnesium oxide nanoparticles (nMgO), with the advantages of non-toxicity, environmental friendliness, ease of availability, and biocompatibility with human cells, are recognized as safe disinfection agents by the U.S. Food and Drug Administration without any harmful byproducts; thus, they hold great promise in both medical therapeutics (Chalkidou et al., 2011; Krishnamoorthy et al., 2012) and water decontamination (Purwajanti et al., 2015). *In vitro* studies have demonstrated that nMgO could be applied as a microbicide against gram-positive (*Bacillus subtilis* and *Staphylococcus aureus*) and gram-negative (*Escherichia coli*) bacteria (Stoimenov et al., 2002; Makhluf et al., 2005) and fungal pathogens (Parizi et al., 2014). Antibacterial activity has been shown to be affected by the size, pH, concentration, and form of the nMgO (Makhluf et al., 2005; Jin and He, 2011). Unlike for agrichemicals, these toxicity mechanisms are most likely due to direct physical and chemical inactivation during the interaction, avoiding the dissolution of vegetative fungal spores through the secretion of amino acids and malic acid (Montag et al., 2006).

Several studies have suggested that the formation of reactive oxygen species (ROS) and its accumulation in cells seem to be the underlying mechanism of metal nanoparticles against bacterial pathogens, particularly because the generated ROS directly destroy the cell multiplication capacity (Horst et al., 2013; Chen et al., 2014). It is generally believed that microorganism disinfection relies on the direct interaction between nanoparticles and biological cells (Chen et al., 2012; Zhao et al., 2018). In particular, Huang et al., further demonstrated that peptide linkages in the bacterial cell wall are physically damaged by the generation of superoxide ions on the surface of nMgO (Huang et al., 2005). Conceivably, nMgO have enormous potential as antibacterial agents, effectively suppressing agricultural bacterial and fungal diseases caused by *Ralstonia solanacearum*, a medical and foodborne pathogen (Jin and He, 2011; Parizi et al., 2014; Imada et al., 2016; Sierra-Fernandez et al., 2017; Cai et al., 2018a). Nonetheless, investigations on the effects of nMgO on fungal pathogens and on elaborate antimycotic mechanisms have scarcely been reported before (Jin and He, 2011). Inspired by previous studies, we hypothesized that nMgO could be antagonistic to fungi by directly acting on fungal cells.

Most importantly, for the purpose of green and sustainable agriculture, a perfect agricultural microbicide would have no phytotoxicity on plants. Excitedly, foliar application of nMgO as nanoscale fertilizers or light absorption promoters significantly promoted the growth of several crops (Imada et al., 2016; Cai et al., 2018a). Typically, tobacco black shank and black root rot are widespread in Chongqing and significantly decrease tobacco quality and yield (Zhang et al., 2003; Huang and Kang, 2010). In addition, despite the high toxicity of nMgO on several phytopathogens, direct evidence for their role in the successful control of pathogen infection *in vivo* is still limited. Thus, the possibility of using nMgO as fungicidal agents should be explored.

In the present study, deeper insight into the antifungal mechanisms of nMgO against phytopathogenic fungi was investigated in comparison with those of macroscale MgO (mMgO). The first step was to focus on the green synthesis of nMgO using a previous method with modification. The physicochemical properties were characterized through X-ray diffraction (XRD) and X-ray photoelectron spectroscopy (XPS). The particle morphology and dispersibility were observed by transmission electron microscopy (TEM) and scanning electron microscopy (SEM). The size distribution was determined by dynamic light scattering (DLS). The direct-contact damage process was evaluated as a possible antifungal mechanism. The effective inactivation of pathogenic *P. nicotianae* and *T. basicola* was illustrated by inhibiting spore germination, impeding mycelial growth and sporulation, and changing the zeta potential of fungal cells. Then, cell morphological observations when nMgO were used were examined using electron microscopy imaging [SEM/energy-dispersive spectrometry (SEM/EDS) and high-resolution TEM (HRTEM)]. The oxidative stress level was also evaluated. In addition, *in vivo* tests were first employed to further study the control efficacy of nMgO and mMgO on two types of fungal diseases under greenhouse conditions. These observations open a new field of vision for exploring the significant potential of metal nanoparticles as a novel disease management strategy in agricultural applications.

## Materials and Methods

### Characterization of MgO Nanoparticles

According to previous studies, nMgO were prepared by slowly mixing 10 ml of *Carica papaya* L. leaf extract with 50 ml of 0.1 M solution of magnesium nitrate dropwise under vigorous agitation. Then, some white precipitates were observed, which were mainly composed of Mg(OH)_2_. Thereafter, the substance was washed with deionized water by centrifugation at 5,000 rpm for 10 min at least three times in order to remove the remaining impurities. Finally, the obtained precipitate was dried at 100°C and calcinated at 400°C to produce nMgO (Oladipo et al., 2017). The shape of nMgO was determined using TEM/HRTEM performed on a PEI Tecnai G2 F30 field emission instrument with an accelerating voltage of 300 kV. XRD was conducted on an X-ray diffractometer system (D/MAX 2200H, Bede 200, Rigaku Instruments C; Cu Kα radiation, λ = 1.5418 Å) to determine the crystallographic structures of the oxide nanoparticles. XPS was carried out using a Thermo ESCALAB 250 photoelectron spectrometer operated with a twin anode Al Kα X-ray source (1,486 eV, 300 W). The size distribution and zeta potential were measured using DLS in a Zetasizer Nano (Malvern, United Kingdom).

### Fungal Strains and Spore Suspension Cultivation

*Thielaviopsis basicola* and *P. nicotianae* fungi used in the experiment were originally isolated from black root- and black stem-infected tobacco plants in a continuously growing field (10 years) in Chongqing by the Laboratory of Natural Product Pesticide of Southwest University and identified by PCR amplification of 18S rDNA using the oligonucleotides ITS1 and ITS4.

*Thielaviopsis basicola* pellets were grown in potato dextrose agar (PDA) plates in Petri dishes (90 × 15 mm) at 25°C for 15 days until a mycelium developed to the edge of the plate. Five milliliters of sterile-distilled water was added and shaken gently, and then the endoconidium was harvested from the surface of colonies and placed in a sterile beaker.

*Phytophthora nicotianae* inocula were grown on oatmeal agar (OA) medium at 28°C until experimental use. OA medium, composed of 30 g of oatmeal, 15 g of agar, and 1,000 ml of distilled water, was boiled for 1 h for use after filtration through two layers of gauze and sterilization. Specifically, the preparation of high-density zoospore suspensions followed previously reported protocols (Kong et al., 2017). Eight (∼1-cm-diameter) condensed hyphal discs were transferred to fresh 10% V8 juice broth at 23°C for 1 week under dark conditions, followed by inducing sporangia in the light for another 2 days. Then, cultures with numerous sporangia were drenched using 4°C cold, disinfected distilled water for 20 min to exhaustively drag the zoospores; and mycelium aggregates were repeatedly rinsed and eliminated. The obtained two types of spore suspensions were filtered using three layers of gauze to the remove residual mycelium and other growth medium constituents. Both fungal spore suspension concentrations were determined and adjusted to 1 × 10^7^ conidia/ml by a hemocytometer (Hausser Scientific, Horsham, PA, United States).

### Mycelial Growth Inhibition Measurements

As reported in the literature, PDA and OA media are typically used for mycelial growth inhibition assays (Sun et al., 2018). Two kinds of fungal circular cakes with approximate diameters of 6 mm were isolated from the cultured fungal colony. Briefly, the as-prepared inocula were carefully placed in the center of sterilized OA (for *P. nicotianae*) and PDA (for *T. basicola*) plates containing a series of concentrations (0, 125, 250, and 500 μg/ml) of nMgO and mMgO. The medium was sonicated before use. After the Petri dishes were sealed using parafilm, *P. nicotianae* agar plates were kept in an incubator at 28°C for 4 days, and *T. basicola* plates were kept at 28°C for 23 days. The colony diameter was measured every day or every 2 days. The antifungal activities were measured as the mean colony diameter (mm) ± SD for growth inhibition. The inhibition ratios were calculated for each fungal strain. All treatments were carried out in triplicate.

### Sporulation and Spore Germination Suppression Assay

The suppressive effects of nMgO on the sporulation and spore germination of the two fungal strains were assessed by the concavity slide method (Chen et al., 2014). Briefly, fungal conidia were collected as mentioned above. Then, 150 μl of each conidial suspension was blended with the same volume of nMgO or mMgO to obtain a series of tested concentrations (0, 125, 250, and 500 μg/ml). Then, approximately (1.0 × 10^7^) 60 μl of hybrid was drawn on the slide and cultivated at 25°C for 4 h in the case of *P. nicotianae* and for 24 h in the case of *T. basicola* under dark conditions using a thermostatic incubator (Kong et al., 2017). Spore suspensions were placed on slides without the addition of any nanoparticles as a blank control. Conidia were considered to be germinated if the germ tube extended to at least twice the length of the spore itself (Kong et al., 2017). The total number of spores and germinated spores in five randomly chosen areas was counted on each slide under a light microscope (Carl Zeiss, Germany) for *P. nicotianae* and *T. basicola*, respectively, in triplicate. Finally, the spore germination rate was determined as the ratio of the average number of germinating conidia to the sum of spores.

As mentioned above, appropriate inducing sporulation medium (ISM) was mixed with 1,000 μg/ml of nMgO or mMgO to obtain final tested concentrations of 0, 125, 250, and 500 μg/ml. The control group comprised only ISM. After sterilization at 121°C for 40 min in an autoclave, 4 ml of the mixture was poured slowly on the circular Petri dish and cooled. Afterward, several *P. nicotianae* and *T. basicola* inocula (∼1-cm-diameter) cultivated on agar medium were transferred into the treated medium. All the culture dishes were placed in a portable thermostatic incubator at 26°C for 24 h with constant illumination to promote sporangium development. Following cultivation, sporangia were observed using an optical microscope (Carl Zeiss, Germany).

### Morphological Changes Observed by Electron Microscopy

Furthermore, the antifungal efficiency of nanomaterials was measured by observing fungal hyphal morphological characteristics after direct attachment. *T. basicola* and *P. nicotianae* zoospores were cultivated in PDA and OA media, respectively, for 24 h at 28°C to form a mycelium. Then, the mycelia were transferred into fresh medium containing 250 μg/ml of nMgO and mMgO, and the samples were grown under the same conditions for another 24 h. Then, the mycelia were collected by centrifugation at 4,000 rpm, immersed in 0.05% Evans blue (EB) dye solution, a kind of azo dye that can penetrate damaged cell membranes and bind with intracellular protein, and stained for 5 min. After being washed at least three times with deionized water, the mortality of hyphal aggregates was observed using an optical microscope (Carl Zeiss, Germany).

Scanning electron microscopy and TEM imaging was applied to observe the morphology and ultrastructure of fungi when treated with nanoparticles. The pretreatment assay was performed as follows. In brief, nanoparticle-treated and untreated *T. basicola* and *P. nicotianae* mycelia cultured on OA medium were collected and then quickly fixed with 2% glutaraldehyde for 2 h at 4°C. Samples were then washed with phosphate-buffered saline (PBS; 0.5 M, pH 6.5) several times. Subsequently, the hyphal samples were subjected to dehydration with a gradient series of ethanol (30, 50, 70, 80, 90, and 100%) for 10 min. After being air-dried naturally, the samples were conductively coated by gold sputter (<10 nm) and submitted to SEM (FEI Quanta 200, Netherlands) operated at accelerating voltages at 30 kV. Additionally, the dried hyphae were embedded in epoxy resin, cut into ultrathin sections, and poststained. Afterward, samples were mounted on a copper grid to examine using TEM (JEOL JEM-1230, Japan) at an accelerating voltage of 180 kV.

### Zeta Potential Determination on the Cell Surface

To record the direct interaction between fungal *T. basicola* and *P. nicotianae* and nMgO and mMgO, equal volumes of overnight-cultured spore suspensions in medium were coincubated with nMgO or nMgO at different concentrations at 28°C. Thereafter, the zeta potentials of the fungal suspension–nMgO mixture, the nMgO suspension dispersed alone in the medium, and the suspension of fungal cells alone were all determined using a ZetaPlus Zeta Potential Analyzer (Malvern, United Kingdom).

### Oxidative Stress Assay

To detect the oxidative stress in fungal hyphae induced by nMgO, we focused on an assay to study the level of ROS in sabouraud liquid medium (SLM), a typical oxidative composite such as hydrogen peroxide (H_2_O_2_) and superoxide anion (O^2–^). A specific non-fluorescent probe, the cell-permeable, non-polar molecule 2’,7’-dichlorodihydrofluorescein diacetate (H_2_DCFH-DA), was utilized in this experiment, which was deacetylated to 2’,7’-dichlorodihydrofluorescein (DCFH) by intracellular esterases once inside the cells (Rispail et al., 2014). DCFH was then oxidized non-specifically by ROS produced in cells to form dichlorofluorescein (DCF), which excited detectable green fluorescence. Briefly, hyphae were cultured in SLM containing 250 μg/ml of nMgO (at the highest concentration) for 24 h at 28°C. After that, hyphae were collected by centrifugation, removing the media. The cells were washed with PBS at least three times. Then, 500 μl of suspensions were suctioned out and mixed with 40 μmol/L of H_2_DCFH-DA in the dark. Then, the treated samples were incubated at 28°C for 90 min, 4 h, and 30 min with moderate shaking. Finally, intracellular ROS generation was quantitatively measured by a fluorescence spectrometer (FluoroMax-4; Horiba Scientific, Tokyo, Japan) with filters for excitation at 485 nm and emission at 525 nm. For visual observation of ROS production, after pretreatment as above, the stained cells were directly placed onto a microscope slide and observed by inverted fluorescence microscopy.

### Plant Material and Inoculation With Phytopathogens

A tobacco cultivar (Yunyan 87) susceptible to soilborne bacterial and fungal pathogens was cultured in an artificial growth climate incubator for 40 days, in which the temperature was set at 30 ± 1°C/28 ± 1°C day/night temperature coupled with a relative humidity of 85–90% and a light period of 14 h, until the fourth leaf was developed.

The two fungal pathogens *P. nicotianae* and *T. basicola* were previously cultivated on the specific agar plates mentioned above, the inoculated conidiospores were harvested, and the inoculated concentration was determined to be 10^7^ spores/ml. Pathogenic infection was performed by root irradiation. Specifically, 20 ml of conidiospore suspension (10^7^ spores/ml, in the corresponding media) was poured around the stem base in separate trials. After inoculation for 24 h, the same volume of the tested concentration of nMgO or mMgO was poured according to the procedure above. The treated tobacco plants were then placed in the climate incubator. Control treatments were conducted with only sterilized water. Twenty uniform tobacco seedlings were chosen for one treatment. Disease symptoms mainly included wilting and stem cankers. After the appearance of the first wilted leaf, the number of infected plants and symptomless plants was recorded for each treatment every day until complete death in the control samples, which occurred 10 days post-inoculation (dpi) for *P. nicotianae* and 15 dpi for *T. basicola*. Disease incurrence was calculated, and controlling efficiency was assessed in terms of morbidity and mortality. All trials with different concentrations of nMgO and mMgO were repeated at least three times.

### Statistical Analysis

Each experiment was conducted with a randomized design. All the experimental data were presented as the mean of three individual observations with standard deviation (SD) and analyzed by SPSS software. For all analyses, significance was calculated using Student’s *t*-test, and differences were considered statistically significant at *p* ≤ 0.05.

## Results and Discussion

### Characterization of MgO Nanoparticles

**FIGURE 1:**
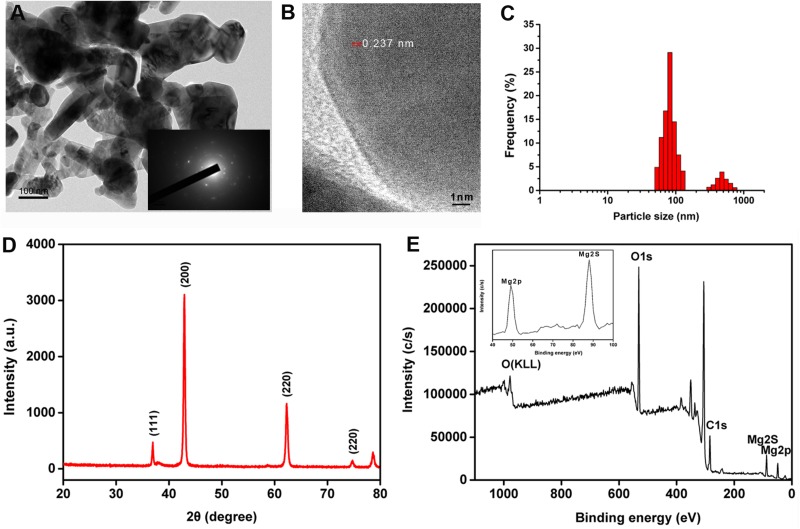
**(A)** Representative transmission electron microscopy (TEM) images of prepared MgO nanoparticles (nMgO) with selected area electron diffraction (SAED) patterns in the inset. **(B)** High-magnification view of nMgO. **(C)** Size distributions of nanoparticles. **(D,E)** X-ray diffraction (XRD) and X-ray photoelectron spectroscopy (XPS) survey spectrum of nMgO. The inset plot illustrates the high-resolution XPS scan spectrum over the Mg 2p and Mg 2s spectral regions of the nanoparticles.

The nMgO were characterized by several technologies, which were applied to analyze the morphological structure and aggregation state. Figure 1A shows that the nanoparticles were irregularly spherical with an average size of 100 nm. However, the nanoparticles tended to agglomerate by forming stacks, where TEM images show the nanoparticle morphology, indicating unsatisfactory dispersibility due to the van der Waals (vdW) force (Stabryla et al., 2018). The inset of Figure 1A shows the selected area electron diffraction (SAED) pattern of nMgO, confirming the nanocrystalline nature and ability to be indexed to the cubic phase nMgO, which is in accordance with the XRD pattern (Makhluf et al., 2005). The lattice fringes in the HRTEM image are separated by an interplanar distance of 0.237 nm (Figure 1B). A typical XRD pattern clearly showed that only several sharp peaks located at 2θ of 36.95°, 42.92°, 62.30°, 74.76°, and 78.61° were assigned to the (111), (200), (220), (311), and (222) crystallographic planes of the face-centered cubic (FCC)-structured MgO nanopowders [Joint Committee on Powder Diffraction Standards (JCPDS) file no. 89-7746] (Figure 1D). No other peaks were detected in the XRD spectrum, indicating the high purity of the obtained nMgO (Ding et al., 2001; Kumar and Kumar, 2008).

X-ray photoelectron spectroscopy measurements were applied for surface analysis and the metal oxidation state. As expected, the peaks observed in the XPS spectra confirmed the increase in the Mg (2p and 2s), C 1s, and O 1s intensities (Figure 1E). Two peaks with binding energies at 368.1 and 374.1 eV in the XPS spectrum were ascribed to Mg 2p and Mg 2s photoelectrons, respectively, which was in agreement with a previous study (Ding et al., 2001). In particular, the C 1s (binding energy at 284.7 eV) and O 1s (binding energy at 531.7 eV) in the XPS spectrum could still be observed.

### Evaluation of the Antifungal Activity of MgO Nanoparticles and Macroscale MgO *in vitro*

**FIGURE 2:**
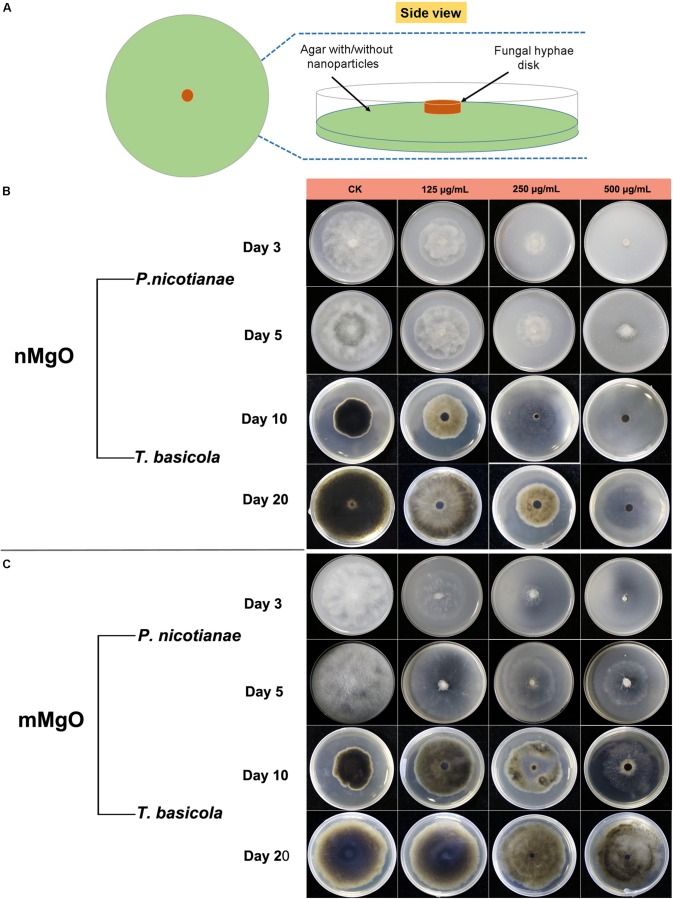
**(A)** Schematic illustration of an antifungal assay. The concentration-dependent inhibitory effects of MgO nanoparticles (nMgO) **(B)** and macroscale magnesium oxide (mMgO) **(C)** on the growth of *Phytophthora nicotianae* and *Thielaviopsis basicola* mycelium transferred onto potato dextrose agar (PDA) and oatmeal agar (OA) media without and with different concentrations (125, 250, and 500 μg/ml) of nanoparticles or bulk materials, respectively. Representative images of agar plates were photographed after incubation for 3 and 5 days for *P. nicotianae* and 10 and 20 days for *T. basicola*.

**FIGURE 3:**
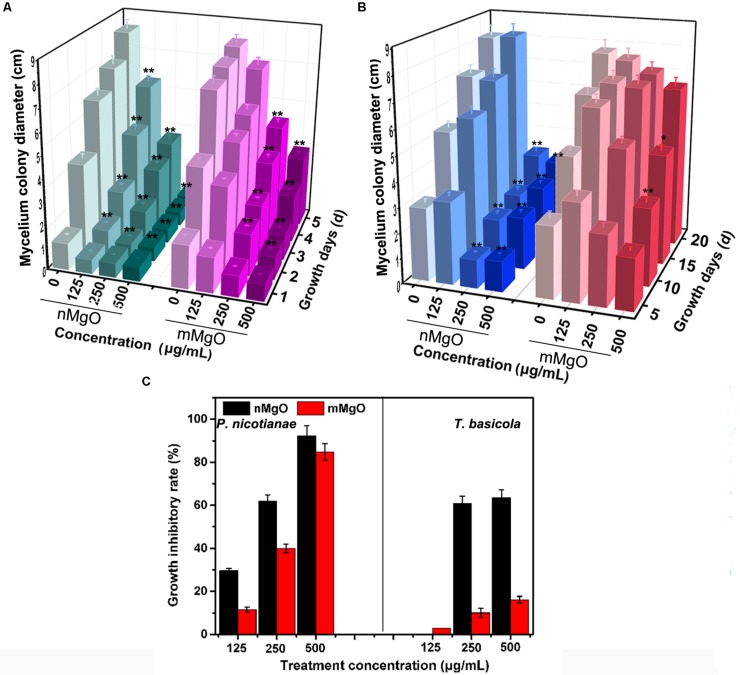
Mycelium colony diameter of *Phytophthora nicotianae* **(A)** and *Thielaviopsis basicola* **(B)** after exposure to potato dextrose agar (PDA) and oatmeal agar (OA) media containing different concentrations (0, 125, 250, and 500 μg/ml) of MgO nanoparticles (nMgO) or bulk materials for 5 and 20 days, respectively. **(C)** Mycelium growth inhibitory rate of two kinds of fungi under deionized water, nMgO and macroscale magnesium oxide (mMgO) treatments. Values are averages, the error bars indicate one standard deviation (*n* = 3), and * and ** on bars indicate significant differences among treatments (*p* = 0.05 and *p* = 0.01).

The antifungal activity was explored by investigating the vegetative growth of *T. basicola* and *P. nicotianae in vitro* under different concentrations of nMgO and mMgO suspension treatments, which is considered an efficient method for evaluating the fungitoxic activity of nanomaterials (Patra et al., 2012). Thus, the antifungal activity of the nanoparticles was evaluated mainly by inactivating hyphal growth, interfering with sporulation formation, and hindering conidiospore germination during the direct interaction between nMgO/mMgO and fungi. To observe the hyphal growth kinetics when interacting with nMgO and mMgO, continuous measurements were carried out during the experimental period. After two kinds of fungal cakes were grown on untreated and corresponding nMgO- or mMgO-containing agar medium at 28°C, the diameter of the mycelial colony was measured. Visually, both fungi treated with increasing concentrations of nMgO showed a progressive inhibitory effect, and agar plate colony images are presented in Figure 2. Over 5 and 20 days of incubation *in vitro*, as illustrated in Figure 2B, it was found that nMgO restrained the mycelial growth of both fungi under all test conditions, displaying dramatic concentration-dependent toxicity effects, which was in accordance with other metal nanoparticles (Rodriguez-Gonzalez et al., 2016; Sun et al., 2018). As the incubation time increased, the mean mycelial diameter of colonies cultivated on plates containing 125–500 μg/ml of nanoparticles was 2.1, 1.32, and 0.63 cm on the third day and 5.84, 3.17, and 0.63 cm on the fifth day for *P. nicotianae*; these values were much shorter than those for the control group (Figure 3A). The values of the untreated samples were up to 6.21 and 8.3 cm after the equivalent intervals.

**FIGURE 4:**
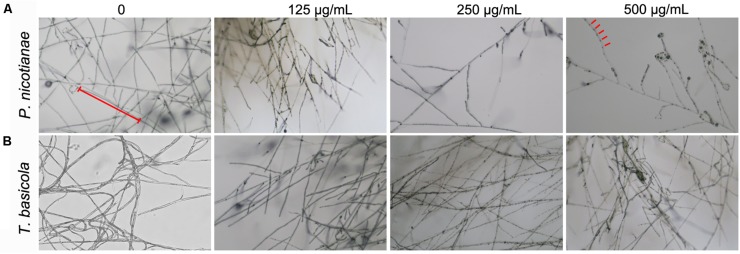
Microphotograph of hyphae of **(A)** *P. nicotianae* and **(B)** *T. basicola* cultivated on various concentrations of MgO nanoparticles (nMgO)-treated potato dextrose agar (PDA) and oatmeal agar (OA) media for 5 and 20 days, respectively.

Similarly, *T. basicola* mycelia were arduously developed in comparison with the control, and filamentous colony growth was clearly inhibited after incubation for 10 and 20 days, displaying 1.89 and 3.18 cm when 250 μg/ml was added and 2.09 and 2.96 cm when 500 μg/ml of nMgO was added (Figure 3B). Although 125 μg/ml of nMgO had no significant impact on the colony diameter, a loosened mycelial structure was observed compared with thick and dense colony in the control group through careful observation (Supplementary Figure 1). After incubation for 5d and 20d, the hyphae of both fungi were develop slowly (Figure 4).

Comparatively, we studied the biocidal activity of mMgO through the same procedure (Makhluf et al., 2005). The results showed that mMgO displayed a trend similar to that observed for the two types of fungi under nMgO treatment, which was concentration dependent (Figure 2C). Remarkably, the growth of *P. nicotianae* hyphae was hindered to a great extent, but for *T. basicola*, the toxicity was moderate. There were no significant differences between the diameters of the two hyphal colonies grown on medium containing 125 and 250 μg/ml mMgO for 10 and 20 days and those of the control, but with thinner mycelia than in the control, especially for *P. nicotianae.*

As shown in Figure 3C, as the concentration increased, the growth inhibition rates of *P. nicotianae* and *T. basicola* reached 29.63, 61.8, and 92.4% and 0, 60.88, and 63.59% after nMgO treatment for 5 and 20 days, respectively, whereas mMgO treatment induced 11.46, 40.0, and 84.80% and 2.91, 10.00, and 16.10% suppression rates, respectively. That is, the growth suppression effect of nanoparticles was heightened gradually when the incubation time was prolonged. It can be speculated that once the original normal hyphae contacted the nMgO, they were greatly damaged, and then, the impaired fungal hyphae continued to grow, but the growth rate was obviously reduced. It seems that the nanoparticles gradually decreased the antifungal activity during the incubation time. Importantly, the high antifungal activity of nMgO occurred in a dose-dependent manner, which is consistent with other metallic oxide nanoparticles and carbon-based nanomaterials (Chen et al., 2014, 2016b).

Again, it is important to mention that the fungistatic activity of mMgO was not as high as that induced by nMgO. It was interesting to observe that compared with their bulked equivalents, metal oxide nanoparticles have proven to exhibit stronger toxicity on bacteria, fungi, and plants (Heinlaan et al., 2002). Notably, compared with their macroformation, ZnO, CuO, and TiO_2_ nanoparticles have shown distinguished antifungal activity toward a large number of phytopathogens, such as *Lycopersicon esculentum*, *Fusarium oxysporum* (Ouda, 2011), *Gloeophyllum trabeum* (Terzi et al., 2016), *Tinea versicolor* (Terzi et al., 2016), *Botrytis cinerea* (Rodriguez-Gonzalez et al., 2016; Hao et al., 2017), and *Pseudoperonospora cubensis* (Cui et al., 2009), suppressing mycelial growth.

This phenomenon is likely attributable to the incremental specific surface area, namely, smaller size, which increases the likelihood of nanomaterials coming into contact with biological samples, facilitating a wide range of complex interactions in nanobiosystems (Jiang et al., 2008). Another reason behind this is that a series of cell-nanoparticle interfaces formed during the nanoparticles interacted with biological cells and membranes, where protein corona formation, particle wrapping, and even intracellular uptake occur (Nel et al., 2009).

### Conidial Spore Germination and Sporangium Formation Repression

Spores function as a minute propagative unit of fungal pathogens, greatly contribute to their pathogenic success on host plants, and possess moderate survival capacity in a dormant state (Judelson and Blanco, 2005). That is, the germination of spores, the first crucial step in vegetative and reproductive protonema development, is an extremely indispensable event. Spore germination and mycelium sporulation can respond sensitively to environmental changes and nutritional stress, which involves cellular morphological and structural alterations as well as metabolism.

**FIGURE 5:**
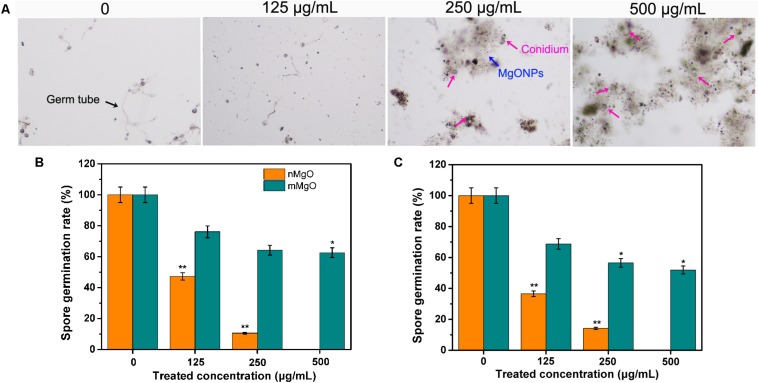
Effects of MgO nanoparticles (nMgO) and macroscale magnesium oxide (mMgO) on fungal conidial germination. **(A)** *P. nicotianae* spores in controls and exposed to nMgO imaged under an optical microscope. Spore germination rate of *P. nicotianae* **(B)** and *T. basicola* **(C)** after 4 and 24 h of direct incubation with sterile water, nMgO and mMgO in the dark, respectively. Values are the mean of three replicates.

In such cases, to further evaluate the fungicidal activity of nanomaterials, we investigated the conidial sporulation of these filamentous fungi in the presence of nMgO and mMgO in the following trial. After incubation with different concentrations, the optical microscope images of *T. basicola* and *P. nicotianae* spore suspensions displayed a distinct reduction in spore germination rate in comparison with that of the untreated fungus acting as the control samples (approximately complete germination). Supplementary Table 1 displays the number of germinated spores of *P. nicotianae* and *T. basicola* after different treatments. Without exception, as shown in Figure 5, a significant statistically reduction (*p* < 0.05 and *p* < 0.01) in the germination rate (46.64 and 10.6% for *T. basicola*, and 36.5 and 14.28% for *P. nicotianae*) under dark incubation was observed in the 125 and 250 μg/ml nMgO groups. When the fungal spores were incubated at the highest concentration, there was no germination, possibly displaying complete sporicidal effects. In contrast, regarding mMgO, the spore germination rate was reduced by 72.1, 36.2, and 22.6% for *T. basicola* and 58.7, 36.5, and 19.0% for *P. nicotianae* in comparison with the control group. This shows that nMgO exert more potent sporicidal activity than do mMgO, and this antagonistic effect on spore germination is as prominent as that of mycelial growth (Figure 2).

**FIGURE 6:**
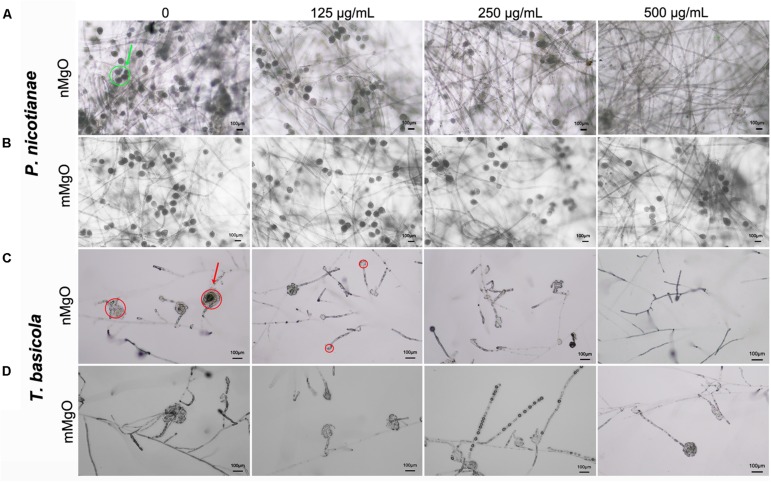
Microscopic images of sporangia of *P. nicotianae* **(A,B)** and *T. basicola* **(C,D)** when they were directly cocultured with the tested concentration of nMgO and mMgO for the corresponding time, respectively.

At the same time, sporangium production was greatly affected in the case of nMgO. As shown in Figure 6, the sporangia in *T. basicola* and *P. nicotianae* grown in the control group had abundant conidia. However, for the nMgO-exposed group at the tested concentration, the decreases in sporangia number and morphology pattern for both fungal pathogens were prominent, which might be due to the presumption that the intensely direct nanoparticle-hyphae interaction disturbed the cell surface protein structure and chemical properties, some of which are involved in sporangium formation (Latijnhouwers and Govers, 2003). Most importantly, the outer electron-dense layer of the *T. basicola* sporangial wall had disappeared, and the structure of the sporangium had become loosened (red arrow). Moderate significant inhibition of sporangia formation was observed even after 500 μg/ml mMgO exposure, which indicated that mMgO had a minor fungistatic effect (Figure 6).

According to published studies, the sporicidal properties of nanoparticles on various phytopathogenic fungi *in vitro* and *in vivo* have been widely reported, involving metals, metal oxide nanoparticles, single-walled carbon nanotubes (SWCNTs), multi-walled carbon nanotubes (MWCNTs), and GO (Dong et al., 2013; Chen et al., 2016b; Liu et al., 2017). A similar work has been reported by Wani and Shah (2012) where nMgO displayed nanotoxicity on several agricultural pathogenic fungi and significant inhibition of the germination of spores of *Alternaria alternata*, *F. oxysporum*, *Rhizopus stolonifer*, and *Mucor plumbeus*. Recently, nMgO were investigated to reduce the spore germination of seven kinds of rot-causing fungi (*Penicillium expansum*, *Aspergillus niger*, *A. alternata*, *M. plumbeus*, *Penicillium chrysogenum*, *Trichothecium roseum*, and *Rhizoctonia solani*) to different degrees without any explanation for this phenomenon. Additionally, Malandrakis et al. (2019) conducted a comparative toxicity assay regarding the antifungal activity of metal nanoparticles against seven types of important foliar and soilborne plant pathogens, such as *A. alternata*, *B. cinerea*, *Fusarium solani*, *Monilinia fructicola*, and *Verticillium dahliae*. Interestingly, copper nanoparticles (CuNPs) were most effective among the NPs on the majority of tested fungal spores, followed by ZnO nanoparticles (ZnONPs), both of which displayed higher toxicity than a commercial fungicide Cu(OH)_2_ (Malandrakis et al., 2019). However, there was no clear indication of their antifungal mechanisms. In this regard, we have found that nMgO could prevent fungal asexual reproduction, and how the nMgO successfully cause great sensitivity to fungal cells will be investigated in subsequent studies.

### Direct Physical Interaction Between Nanoparticles and Fungal Cells

Many studies have reported the direct interaction between various kinds of engineered nanomaterials and biological samples, including bacteria, fungi, and cells, as well as the surface adhesion and cellular uptake patterns (Bhabra et al., 2009; Rodriguez-Gonzalez et al., 2016). As expected, some metal oxide nanoparticles dock on the surface of pathogenic bacteria (Jiang et al., 2009). To characterize the effects of nMgO on fungal hyphae, we monitored the morphological changes in live cells by SEM/EDS to visually observe the presence of nanoparticles on the hyphae.

**FIGURE 7:**
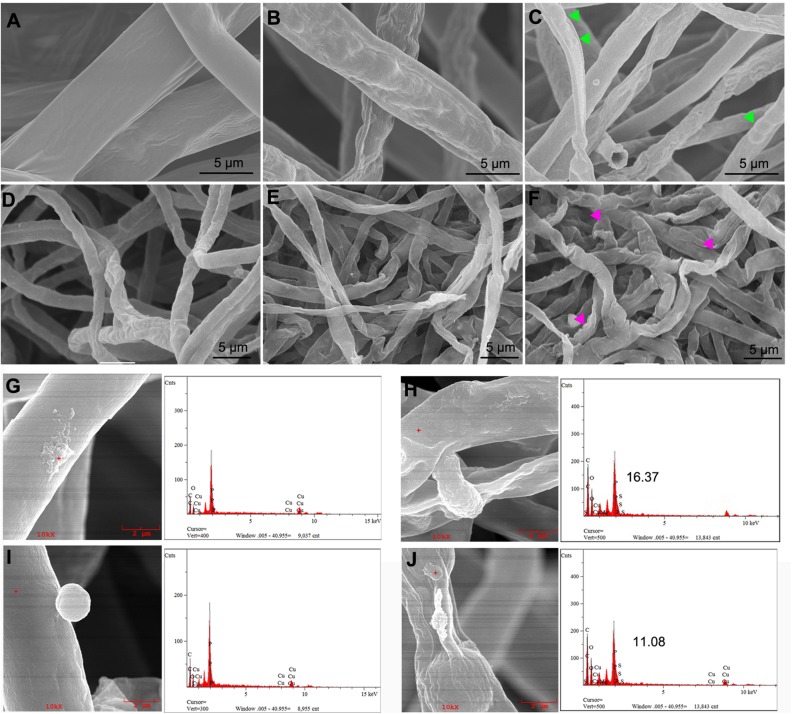
Scanning electron microscopy (SEM) observations of fungal hyphal morphological changes directly exposed to nanoparticles. *Thielaviopsis basicola* and *Phytophthora nicotianae* cells were coincubated with sterile water **(A,D)**, 500 μg/ml of macroscale magnesium oxide (mMgO) **(B,E)**, and MgO nanoparticles (nMgO) **(C,F)** and then submitted to SEM. After direct attachment to nMgO, both hyphae suffered from malformed structure swelling (see the arrowheads in panels **C,F**). The attachment of nMgO to the cell wall of *P. nicotianae* and *T. basicola* after incubation without and with nMgO (500 μg/ml) was detected by SEM/energy-dispersive spectrometry (EDS), respectively. *P. nicotianae* **(G)** and *T. basicola* **(I)** hyphae without aggregates adhered and the nMgO attached to the surface of the hyphae **(H,J)**. The corresponding EDS spectra represent the qualitative elemental analysis of the nMgO.

Two types of vegetative mycelia prepared in this experiment were incubated for 3 h with various concentrations of nanoparticles and then supported on the grid and observed. Compared with the untreated samples, as shown in Figures 7A,D, which maintain a full, uniform, and well-developed tube-like structure, treatment of *T. basicola* and *P. nicotianae* with nMgO at 500 μg/ml led to obviously unfavorable changes following collapsed morphologies under SEM after exposure (Figures 7A,B). The mycelia became sunken and swollen, with an abnormal structure. Furthermore, EDS was applied to determine whether nMgO were present in or on fungi and to verify the chemical nature of the attached agglomerated particles, as this technology could highly monitor the atomic number of each atom present in the material (Rodriguez-Gonzalez et al., 2016). Additionally, the existence of nMgO on the hyphal surface was clearly confirmed, causing local impairment of cell membranes (Figure 7). Precisely, nMgO embedded in the cell membrane were even monitored. These observations provide support for particle-specific contributions to the antifungal mechanisms of metal-based nanoparticles (Stabryla et al., 2018).

**FIGURE 8:**
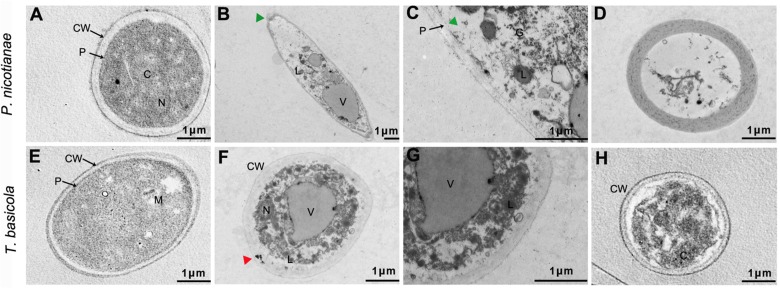
Typical transmission electron microscopy (TEM) ultrastructural micrographs of *Thielaviopsis basicola* and *Phytophthora nicotianae* hyphae incubated with water (control), 500 μg/ml of MgO nanoparticles (nMgO) and macroscale magnesium oxide (mMgO) for 2 h. Hyphae interacted with nMgO and then fixed by vitrification before bright-field TEM analysis. **(A,E)** Untreated hyphae with regular cell wall outline and plasmalemma are clearly visible. **(B,F)** Hyphae exposed to nMgO, along with partial cell wall injury, plasmalemma disappearance, disorganized cytoplasm, and vacuolation (green triangular arrows). **(C,G)** correspond to the magnifications of panel **(B,F)**, showing aggregates of glycogen granules. **(D,H)** Hyphae treated with mMgO having integrated cell wall and plasmalemma while undergoing some injury indicated by cytoplasmic disorder. CW, cell wall; P, plasmalemma; C, cytoplasm; G, glycogen granules; L, lipid droplets; M, mitochondrion; N, nucleus; V, vacuoles; Bar = 1 μm.

In addition, TEM images showed that the control fungal mycelia involved the regular inner and external layers of the cell wall and a normal dense cytoplasm, where a regular disposition of organelles was clearly visible (Figures 8A,E). However, 500 μg/ml of nMgO-treated samples displayed tenuous and distorted sensitive inner membranes, with only the external layer of the partially damaged cell wall (green triangular arrows) (Figures 8B,F). Other anomalies, an increased aggregation of glycogen granules, several intracellular vesicles, and vacuoles in the cytoplasm, were clearly observed (Figures 8C,G). For mMgO, an intact cell envelope under cytoplasmic disorder was observed, especially in *P. nicotianae* cells, with hardly recognizable organelles (Figures 8D,H). It seems that the nMgO inactivate the fungi by injuring the cell plasmalemma originating from the direct physical interaction, as in bacteria (Cai et al., 2018a). This means that the inactivation of hyphae was ascribed to some irreversible toxicity behaviors caused by nMgO, including vacuolation and disorganized enterocytes, which are associated with nanoparticle attachment and penetration, as represented by the triangle arrowhead in Figures 8C,G. In contrast, fungal cells have a normal structure and not quite severely inordinate cytoplasm. It can be assumed that, in brief, the first step may be local damage to the surface cell wall and subsequent destruction of the plasmalemma; thus, a series of important reactions occurs, including subsequent nanoparticle absorption and interaction with cellular macromolecules such as DNA, protein, and lipids, inducing cell death (Patil et al., 2007; Bhabra et al., 2009).

Generally, the inactivation effects of nanoparticles are influenced by aggregation conditions, geometry, size, and physical properties (Herd et al., 2013). Several reports have studied metal nanoparticles that physically covered and penetrated the bacterial cell wall and revealed a tendency different from that of their microscale aggregates, such as SiO_2_ and Al_2_O_3_ (Jiang et al., 2009; Xue et al., 2014; Rodriguez-Gonzalez et al., 2016). It seems that the mechanistic interfacial interaction between the biological membrane and nanoparticle is crucial to understand the underlying mechanism (Krishnamoorthy et al., 2012; Sharma et al., 2015). These phenomena might be associated with the cell wall composition and structure of the fungus. The hyphal cell wall comprises a mixture of chitin, β-1,3 glucans, and β-1,6 glucans and a wide variety of glycoproteins (Judelson and Blanco, 2005; Brown et al., 2015). In particular, certain glycoproteins with adhesive functions, also known as adhesins, participate in adhesion to organic and inorganic surfaces and are involved in host–pathogen interactions (Bamford et al., 2015). Two major members include glycosylphosphatidylinositol (GPI)-modified cell wall proteins and agglutinin-like sequence (ALS) families (Dranginis et al., 2007; Bamford et al., 2015). In particular, nanoparticles sometimes act as supporters facilitating direct contact, similar to carbon nanotube (CNT)-induced pathogen aggregation interactions (Gu et al., 2005). CNTs are appropriately functionalized with sugar-based ligands that can be recognized by receptors on the surface of *Bacillus* spores (Luo et al., 2009).

### Destabilization of Fungal Cell Membrane

**FIGURE 9:**
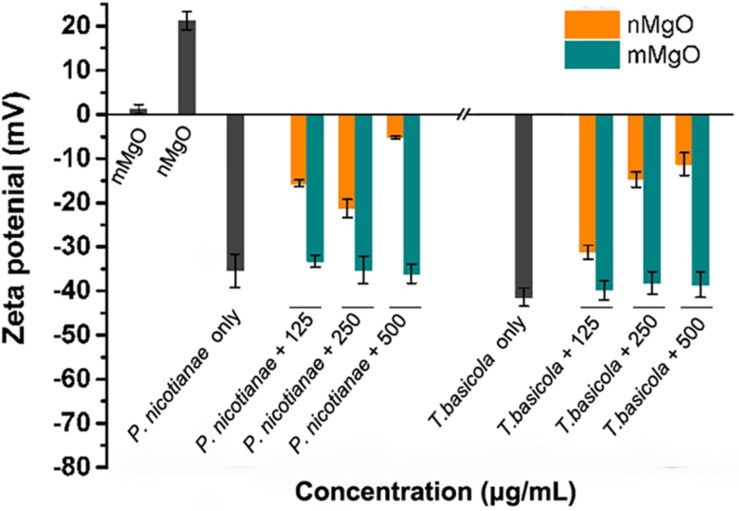
Fungal cell membrane potential changes after exposure to various concentrations of MgO nanoparticles (nMgO) and macroscale magnesium oxide (mMgO).

Furthermore, it cannot be ignored that the fungal cell wall is negatively charged because of the contribution of glycoproteins. The electrostatic interaction might mediate the excellent nanoparticle–cell aggregates, which can be observed in previous reports regarding the antibacterial activities of a series of nanoparticles (Chen et al., 2016a, b; Pan et al., 2013). Indeed, our previous studies have found that nMgO and graphene were visibly directly attached to phytopathogens, affecting energy metabolism and cell membrane potential (Cai et al., 2018a). Ideally, adsorption of nMgO on fungal cells could disturb membrane potential as a result of enhanced adherence. To test this hypothesis, the zeta potential of fungal cells in the presence of nanoparticles was further investigated. As shown in Figure 9, after exposure to nMgO, both fungal cells carried lower negative charges than their highest values, whereas all the fungal samples treated with mMgO showed relatively similar negative charges to the untreated samples. Presumably, the electrostatic interactions between positively charged nMgO and fungi changed the zeta potential of fungal cells, resulting in the close contact of nMgO and deposition on the cell surface (Pan et al., 2013). Enhanced adherence to pathogens of antifungal drugs was caused by decreased electric repulsive forces (Miyake et al., 1990). Consequently, nanoparticles could be capable of physically damaging the cell envelope, which was confirmed by SEM and TEM imaging above (Sharma et al., 2015; Cai et al., 2018a).

Analogously, the destabilization of the cell outer membrane in gram-negative bacteria was observed through the accumulation of envelope protein precursors after exposure to AgNPs and GO-AgNPs, even in gram-positive bacteria with a strong physical barrier (Chen et al., 2014; Zhao et al., 2018). Previous results, reported by Leung and coworkers, proved that nMgO directly interacted with *Escherichia coli* and caused the downregulation of outer membrane proteins (Omp), such as channel porins and ion channel proteins, as well as disturbance of proteins involved in the membrane lipid metabolism process, making cells liable to lyse (Leung et al., 2014). The strong adsorption of ZnONPs on *Chlorella* sp. cells showed excellent mechanical cell damage, as expected owing to the highly negatively charged algal exudate interaction with the positively charged ZnONPs (Chen et al., 2012). Understandably, nMgO directly contacted the intracellular membrane, forming a highly heterogeneous nanoparticle–cell interface. The nanoparticles then induced dynamic physiochemical interactions driven by adhesion forces, which can stem from either specific or non-specific interactions, such as electrostatic, vdW, and hydrophobic forces, twisting and deforming the membrane, thereby increasing the permeability of the cytoplasmic membrane to nanoparticles (Zhang et al., 2015).

### Oxidative Stress Response in Fungal Cells

Considering the prominent activity of nMgO against fungal cells as a result of the direct interaction, we further investigated whether intracellular and cell surface oxidative stresses are produced, which was supposed to be the most valid mechanism of nanoparticles in biological systems. Previously, we found that nMgO induced the accumulation of ROS in bacterial *Ralstonia solanacearum* cells after treatment with low concentrations (Cai et al., 2018a). This is because free radicals generated by the metal nanoparticles can oxidize bacterial cell membrane lipids (Applerot et al., 2012; Lopes et al., 2016). However, oxidative stress in fungal pathogens has not been studied when they interact with nanoparticles. To our knowledge, the most important indicative factors of oxidative stress burst in biological cells include several species, such as H_2_O_2_, O_2_, and ROS (Leung et al., 2014; Rispail et al., 2014).

**FIGURE 10:**
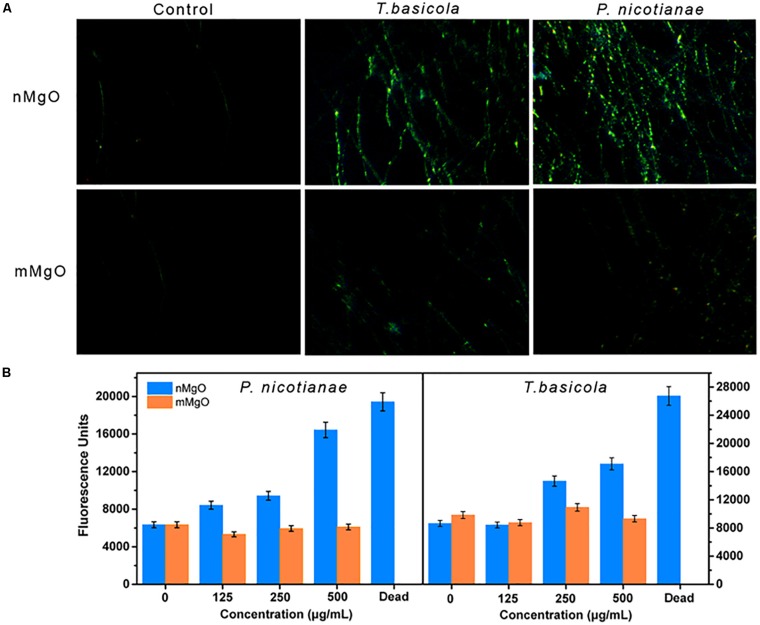
**(A)** Fluorescence microscope observations and **(B)** fluorescence intensity measurements, which show the reactive oxygen species (ROS) level in *Phytophthora nicotianae* and *Thielaviopsis basicola* hyphae under a series of MgO nanoparticles (nMgO) and macroscale magnesium oxide (mMgO) concentration exposures.

Figure 10A shows that H_2_DCFH-DA fluorescence was inordinately produced when two types of fungal hyphae were exposed to a series of nMgO concentrations compared with the control. A higher concentration of nMgO led to superior fluorescence generation, indicating that nMgO indeed triggered the generation of ROS. Interestingly, no significant differences were found when the fungus was incubated with mMgO (Figure 10B). A similar ROS-stimulatory effect was also observed in our previous study regarding the antibacterial activity of nMgO toward *R. solanacearum* (Cai et al., 2018a). Nonetheless, the result is in contrast to the results of one study that assayed nMgO in bacteria where there was no evidence that ROS were detected (Leung et al., 2014). This phenomenon is attributed to the differences in concentration of native defects on the surface of the prepared nMgO under various conditions. In particular, nanoparticles can produce more measurable ROS than their bulk counterparts, likely due to the larger surface areas of the nanoparticles providing more absorption sites for UV irradiation, whereas macro-TiO_2_ and ZnO do not yield any ROS (Li et al., 2012). In either case, it is likely that the surface properties of nanoparticles are responsible for the different behaviors in terms of ROS generation (Leung et al., 2014).

### Efficiently Managing Soilborne Fungal Diseases

**FIGURE 11:**
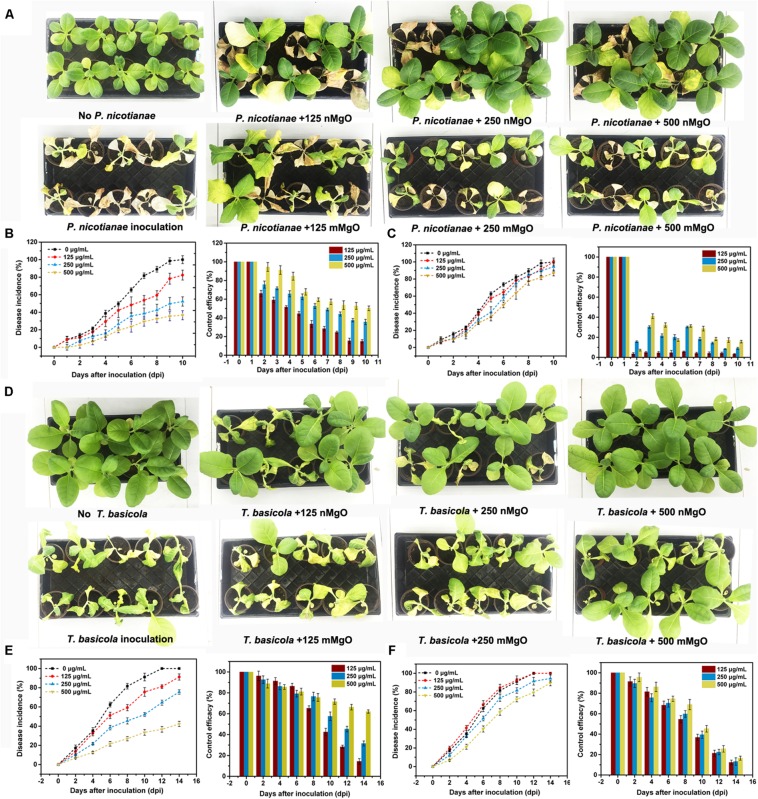
Disease symptoms of representative tobacco seedlings exposed to distilled water (control), MgO nanoparticles (nMgO), and macroscale magnesium oxide (mMgO) at various concentrations of 125–500 μg/ml after inoculation with **(A)** *Phytophthora nicotianae* and **(D)** *Thielaviopsis basicola*. The corresponding disease incurrence and control efficiency when root was irrigated with **(B)** nMgO and **(C)** mMgO for 10 days post-inoculation with *P. nicotianae* and **(E)** nMgO and **(F)** mMgO for 15 days post-inoculation with *T. basicola*.

The control efficacy of nMgO against tobacco soilborne fungal diseases (black shank and black root rot) was first evaluated by pot experiments in terms of disease incidence. Figure 11 shows the two kinds of fungal disease incidence after inoculated tobacco exposure to various concentrations (0, 125, 250, and 500 μg/ml). These results indicated that nMgO exhibited eminent antifungal activity toward *T. basicola* and *P. nicotianae*, decreasing the disease incidence to 91.2, 75.61, and 42.35% and 82.35, 52.78, and 36.58% when compared with 100% for the control, whereas the morbidity of the disease remained high in the case of mMgO exposure, probably owing to the inferior germicidal activity and larger particle size of this bulk metal oxide (Cai et al., 2018a). The control efficiency of *P. nicotianae* and *T. basicola* reached 14.9, 35.6, and 50.2% and 14.35, 31.50, and 62.1% for the different nMgO treatments, respectively. However, the control efficacies of both diseases were below 20% for mMgO application. After incubation for 10 and 14 days at the highest concentration, it was noted that the black shank and black root rot disease wilting symptoms of tobacco exposed to nMgO were prominently reduced in comparison with those of plants watered with water, proving that this type of nanoparticle could serve as a broad biocide for fungal disease management (Figures 11A,D).

Considering the experimental results, there are reasons to believe that nMgO might be very suitable alternatives to fungicides for agricultural disease control. In fact, investigations on the potential of nanomaterials as microbicides have increased in recent years. To a great extent, their biocompatibility and non-toxicity on plants, even human cells, are reasons for pesticide substitutes for plant disease management, especially TiO_2_, MgO, and MWCNTs (Villagarcia et al., 2012; Elmer and White, 2016). In such cases, nMgO have been applied as a microbicide for diversified disease treatments, such as for bacterial wilt (*R. solanacearum*) (Imada et al., 2016) and Fusarium wilt (*F. oxysporum*) (Parizi et al., 2014). In addition, it has been reported that other metallic nanoparticles, such as copper-based nanoparticles, ZnO, and AgNPs, displayed their effectiveness in controlling bacterial and fungal diseases *in vivo* in both pot experiments and field tests, which involved tomato late blight disease (*Phytophthora infestans*) (Giannousi et al., 2013), gray mold (*B. cinerea*) (Rodriguez-Gonzalez et al., 2016; Hao et al., 2017), leaf spot disease (*Xanthomonas perforans*) (Ocsoy et al., 2013), spot blotch disease (*Bipolaris sorokiniana*) (Mishra and Singh, 2015), powdery mildew (*Podosphaera pannosa*) (Hao et al., 2019), tomato Fusarium wilt (*F. oxysporum*) and Verticillium wilt (*V. dahliae*) (Elmer and White, 2016), and Fusarium head blight (*F. graminearum*) (Chen et al., 2016b). TiO_2_ has also drawn on its strengths of photocatalytic disinfection to become a novel approach for the control and inactivation of phytopathogenic fungi, such as Fusarium head blight and tomato gray mold (Paret et al., 2013; Zhang et al., 2013; Rodriguez-Gonzalez et al., 2016). As expected, nanomaterials can be applied as innovative antimicrobial products mainly because their performance is superior to that of some widely commercially used agrochemicals (Giannousi et al., 2013).

**FIGURE 12:**
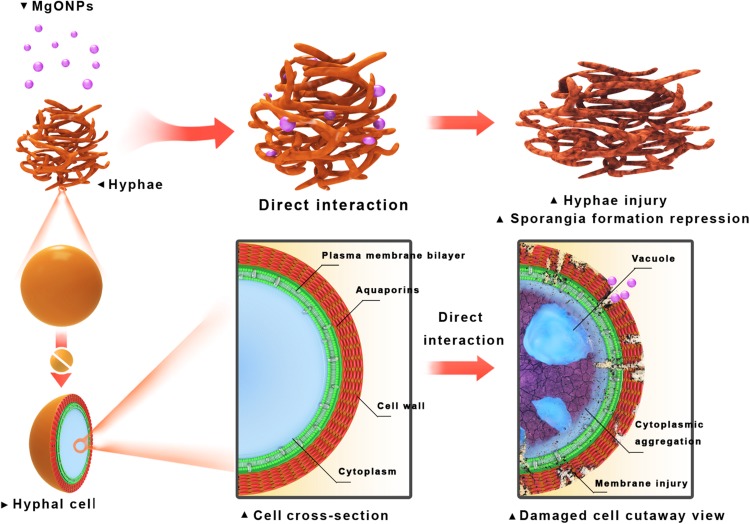
Schematic illustration of inactivation toward fungal pathogen conidia and mycelia through direct interaction with the MgO nanoparticles (nMgO).

Nevertheless, the comprehensive mechanisms by which nanomaterials might inactivate phytopathogens *in vitro* and *in vivo* are not well understood. The tested two kinds of fungi, taxonomically classified as oomycetes, can reproduce asexually through hyphal fragments, sporangia that produce directly and release motile zoospores (Kong et al., 2017). Then, they complete multiple cycles of infection on host plants and spread within a short period of time (Judelson and Blanco, 2005). It is generally demonstrated that the main toxicity mechanisms rely on direct physical interactions and ROS accumulation (Jiang et al., 2009; Li et al., 2012). By comparison, in this paper, we investigated an improved inactivated mode of nMgO rather than mMgO, including suppressing fungal germination and growth, inducing ROS production, destroying membrane integrity, and altering morphological characteristics. We proposed that all the behaviors of nMgO arose from an initial direct contact with fungal hyphae and spores, which have been presented in SEM and TEM images; hence, cellular uptake was observed (Figure 4), as schematically indicated in Figure 12. It seems that this could be explained by the nanosized effects, which are clearly different from those of their bulk counterparts.

In addition, the role of metal ions dissolved from metal oxide nanoparticles should be heavily considered for the high antimycotic activity of nMgO. It has been proven that Mg ions not only interact with cellular proteins and denaturing proteins but also act as essential mineral elements for plant growth under foliar application and soil amendment. This is because Mg participates in numerous direct and indirect specific physiological and biochemical processes owing to the metabolic functions of Mg in plant physiology and consequently plays a crucial role in plant defense mechanisms in biotic stress situations. This may contribute to the high plant disease efficiency (Dordas, 2008). In addition, Mg is also a constituent of structural tissues, such as lignin, suberin, and the middle lamella, together with Ca, which makes some pectic substances more resistant to degradation by pectolytic enzymes of various bacterial and fungal pathogens (Huber and Jones, 2013).

Tobacco, known as an important economic crop, is widely cultivated in southern growing areas in China and has suffered from black shank and black root rot disease in recent years, causing devastating losses of 50–60% and up to 100% (Jiang et al., 2017). Although the reasons for the extensive invasion of soilborne pathogens on hosts are intricate and still unclear, our previous studies speculated that it is commonly the result of soil acidification and excessive fertilizer utilities, which readily lead to micronutrient deficiencies, such as Mg, Fe, and Mn, and low availability of these elements (Li et al., 2017; Shen et al., 2018). Thus, nutrient balance in soil and plants is destroyed, increasing the occurrence and severity of various root and foliar diseases (de Oliveira et al., 2005). Fittingly, low soil pH is conducive for the appropriate dissolution of metal ions, even though nanoparticles aggregate (Bian et al., 2011). It is believed that nMgO could potentially be applied as nanofertilizers for supplemental nutrients in agriculture without any toxicity to plants (Cai et al., 2018b). Moreover, recent studies have shown that, as a result of their unique properties, nanoparticles may influence lettuce (*Lactuca sativa*) metabolic activities, improving and mobilizing phosphorus availability and uptake in the rhizosphere (Zahra et al., 2015; Saharan et al., 2016). Most importantly, the phytotoxicity and biocompatibility of nanoparticles toward plants need to be investigated. Fortunately, we previously found that nMgO enhanced tobacco growth under greenhouse conditions (Cai et al., 2018a). Additionally, there was significant improvement in wheat root growth and grain yield after foliar nano-Mg application under greenhouse conditions. Raliya et al., demonstrated that biosynthesized nMgO improved shoot–root growth (18.2 to 49.2%) and chlorophyll photosynthetic pigment (76.1%) in clusterbean (*Cyamopsis tetragonoloba*) (Raliya et al., 2014). From these perspectives, our study indicates some potential benefits of using nMgO as a non-phytotoxic fungicide despite being applied at higher doses to the matrix. However, the nanoparticle–pathogen interaction in rhizosphere soil and whether the host plant plays an important role in regulating the pathogen invasion process still require more investigation.

## Conclusion

In conclusion, this study demonstrated for the first time the prominent antifungal activity of nMgO against two types of soilborne pathogens, *P. nicotianae* and *T. basicola*, *in vitro* and in a greenhouse compared with that of mMgO. The results illustrated that, in comparison with mMgO, nMgO had a significantly higher inhibitory effect on spore germination, sporangium formation, and hyphal development. All the toxicity behaviors may be mostly attributed to nanoparticle–cell direct contact, classically observed using SEM/EDS and TEM technologies, and the subsequent oxidative stress. Additionally, investigations in greenhouse experiments that irrigated the application of nanomaterials found that nMgO reduced fungal disease occurrence when compared with untreated controls. Our results proposed a new view that nMgO could act as an underlying alternative to fungicides for tobacco black shank and root black rot management, even possibly controlling other plant pathogens. Nevertheless, future investigations are needed to evaluate how the nanoparticles affect the toxic activities of pathogens from the view of the whole genome and clarify the control efficacy under field conditions, as well as the related microbial response mechanism in rhizospheric soils.

## Data Availability Statement

All datasets generated for this study are included in the article/Supplementary Material.

## Author Contributions

JC and WD designed the experiment. LW, ML, and SL performed the experiment. JC and ZL analyzed the experimental data. JC and LW wrote the manuscript. WD revised and polished the manuscript.

## Conflict of Interest

The authors declare that the research was conducted in the absence of any commercial or financial relationships that could be construed as a potential conflict of interest.
