# Supplementary material for: Comparative Study on the Fungicidal Activity of Metallic MgO Nanoparticles and Macroscale MgO Against Soilborne Fungal Phytopathogens
Source: Front Microbiol. 2020 Mar 12;11:365. doi: 10.3389/fmicb.2020.00365 (PMC7080993; doi:10.3389/fmicb.2020.00365)
Supplement: Supplementary file 1 [file Data_Sheet_1.docx]

Supplementary Material

**Supplementary Table 1.** Number of germinated spores of *P. nicotianae* and *T. basicola* strains as obtained from slide bioassay

| Fungal strain | Concentration (μg/mL) | Total number of teated spores  (spore/mL) | Number of germinated spores | |
| --- | --- | --- | --- | --- |
|  |  |  | nMgO  (spore/mL) | mMgO  (spore/mL) |
| *P. nicotianae* | 0 | 63x10^4^ | 63x10^4^ | 63x10^4^ |
|  | 125 |  | 23x10^4^ | 37x10^4^ |
|  | 250 |  | 9x10^4^ | 23x10^4^ |
|  | 500 |  | 0 | 12x10^4^ |
| *T. basicola* | 0 | 75x10^4^ | 75x10^4^ | 75x10^4^ |
|  | 125 |  | 35x10^4^ | 54x10^4^ |
|  | 250 |  | 8x10^4^ | 27x10^4^ |
|  | 500 |  | 0 | 17x10^4^ |


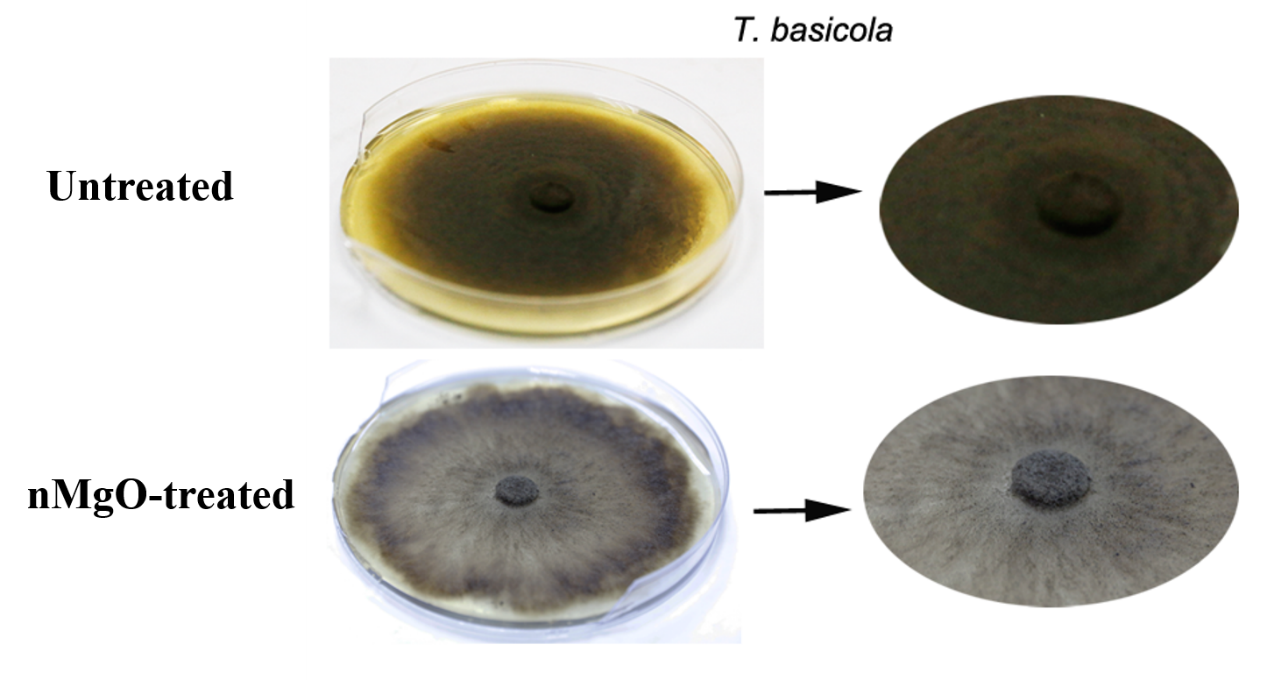


**Supplementary Figure 1.** The mycelium density of *T. basicola* grown on untreated and 125 μg/mL nMgO-treated agar medium.
